# Molecular transmission network characteristics and high-risk transmission analysis of newly reported HIV-1 subjects in Nantong, China

**DOI:** 10.3389/fimmu.2025.1716273

**Published:** 2025-12-08

**Authors:** Dongyang Wang, Xiaoyi Zhou, Tianze Yuan, Hongli Xia, Kai Liu, Anni Chen, Ping Zhu, Xun Zhuang

**Affiliations:** 1 Department of Epidemiology and Medical Statistics, School of Public Health, Nantong University, Nantong, Jiangsu, China; 2 Nantong Center for Disease Control and Prevention, Nantong, Jiangsu, China; 3 Nantong Tongzhou District Center for Disease Control and Prevention, Nantong, Jiangsu, China

**Keywords:** HIV-1, subtype, transmission network, molecular epidemiology, drug resistance

## Abstract

**Introduction:**

Nantong is currently experiencing an HIV-1 epidemic. However, detailed information regarding the local transmission chains remains limited. This study aimed to combine genomic, epidemiological, and spatial data to investigate the genotypes, drug resistance, and transmission patterns of newly reported HIV-1 subjects in Nantong.

**Methods:**

A total of 1619 newly diagnosed HIV-1 cases were identified, of which 1203 valid sequences were included for analysis. The HIV-1 *pol* genes were amplified and sequenced for the analysis of genotype, drug resistance, and molecular transmission network. Logistic regression was used to identify factors associated with being within the molecular transmission networks. Spatial analysis was conducted using intensity matrices of HIV-1 transmission links between regions.

**Results:**

Among the 1,203 subjects, the male-to-female ratio was 4.3:1, with a median age of 49 years (IQR: 33-58). Phylogenetic analysis indicated that CRF07_BC was the dominant strain, followed by CRF01_AE and CRF55_01B. The overall prevalence of pretreatment drug resistance was 9.3%, with V179E as the most common mutation. The molecular transmission network was constructed at a 0.5% genetic distance threshold. Of 1,203 valid sequences, 326 (27.1%) were incorporated, forming 116 clusters ranging from 2 to 17 sequences, including 31 clusters with ≥3 sequences. Multivariate logistic regression analysis indicated that individuals aged 70 years or older (OR=2.17, 95% CI:1.02-4.62), infected with subtype C (OR=1.98, 95%CI:1.11-3.54), CD4+ cell counts of 200-500 cells/μL(OR=1.85, 95%CI:1.29-2.65) and >500 cells/μL (OR=1.87, 95%CI:1.08-3.27) were more likely to be within the molecular transmission networks. Spatial analysis found that the proportion of inter-city transmission was lowest in Rudong (46.8%) and highest in Tongzhou (80.2%).

**Discussion:**

The HIV-1 epidemic in Nantong was characterized by the increasing dominance of CRF07_BC, rising pretreatment drug resistance, and extensive inter-city molecular clustering. Older people, particularly older men, are emerging as a critical population sustaining local transmission. These findings highlight the need for continuous molecular surveillance and region-specific prevention strategies tailored to high-risk groups to mitigate further HIV-1 spread.

## 1 Introduction

The transmission of human immunodeficiency virus (HIV) remains a huge challenge to global public health. By the end of 2024, approximately 1.355 million people were living with HIV/acquired immunodeficiency syndrome (AIDS) in China, with an increase of about 65,000 cases compared to the end of 2023. The situation of AIDS prevention and control in China remains critical, with the number of infections continuing to rise among men who have sex with men (MSM) and older heterosexual individuals (1, 2).

Field epidemiological investigations have certain limitations in tracing HIV transmission patterns. However, the molecular transmission network, based on evolutionary theory and sequence analysis, provides a new perspective for understanding disease transmission. Due to the lack of proofreading ability in HIV reverse transcriptase, the virus genome is highly variable. Each HIV-infected individual carries a unique strain with distinct genetic characteristics. Infected individuals who share a transmission relationship often carry viral strains with high genetic similarity. Phylogenetic analysis based on viral genomic sequences can be used to construct molecular transmission networks among HIV-infected individuals, revealing transmission relationships and patterns at the molecular level, thus providing clues for targeted interventions (3).

In 2009, the United States applied molecular transmission network analysis to AIDS prevention. By integrating molecular surveillance with epidemiological investigations, this approach effectively identified high-risk individuals and demonstrated that two individuals with highly similar viral sequences were likely part of the same transmission cluster (4). Since then, related studies have increased globally (5–7). In 2024, a research team at Brown University developed an automated system that integrates HIV sequence data with clinical records, sociodemographic information, and laboratory data to generate molecular transmission networks. By conducting interviews with individuals identified within these networks, the system was able to uncover additional high-risk individuals and newly diagnosed HIV cases, thereby contributing to the reduction of HIV transmission (8).

Molecular transmission network analysis has also been applied in studies focusing on AIDS prevention in China. A study in Qinzhou reported that older HIV-infected individuals were more likely to form clusters within molecular transmission networks (9). In addition, molecular transmission network analysis have been widely used for monitoring subtypes and drug-resistant variants. In 2024, a study conducted by the Chinese Center for Disease Control and Prevention (China CDC) identified CRF01_AE (38.6%) and CRF07_BC (33.2%) as the predominant circulating recombinant forms (CRFs) in China. Overall, CRFs accounted for 88.5% of all HIV-1 subtypes in the country (10). The emergence of recombinant strains is generally attributed to superinfection or coinfection involving different genotypes within the same host (11, 12). Furthermore, a study conducted in Sichuan Province reported a pretreatment drug resistance (PDR) rate of 8.12% among newly diagnosed HIV-infected individuals. Among them, 37.38% were embedded within molecular transmission networks, and individuals carrying identical drug resistance mutations were often interconnected within these networks (13).

Nantong city is located in eastern China, administering three districts, three county-level cities, and one county, with about 7.7 million population. It is a low prevalence area of AIDS. In 2024, 391 newly diagnosed cases of HIV/AIDS were reported, with older individuals (aged 50 years or older) accounting for 47.8%. Our research focuses on newly reported HIV/AIDS patients from 2021 to 2024, and analyzes the distribution of HIV-1 subtypes, the characteristics of molecular transmission network, and the prevalence of PDR. The aim of this study is to elucidate the transmission patterns among local HIV-infected individuals and provide scientific evidence to support targeted prevention and control strategies for AIDS.

## 2 Materials and methods

### 2.1 Study participants

A total of 1,619 HIV-1 cases were newly diagnosed from January 2021 to December 2024 by the Center for Disease Control and Prevention in Nantong, China. All subjects had not received antiretroviral therapy (ART) at the time of diagnosis. They were recruited in the outpatient service using convenience sampling method. Sociodemographic information (e.g., including age, gender, ethnicity, education level, occupation, marital status), behavioral factors (e.g., transmission routes and high-risk sexual behavior history), and biological factors (e.g., CD4^+^ cell count) were collected through the China HIV/AIDS Prevention and Control Information System. Venous blood samples were also collected, and plasma was isolated and stored at -80°C for subsequent testing.

This study was approved by the Ethics Committee of Nantong Center for Disease Control and Prevention (Approval number: NO. [2024025]).

### 2.2 HIV-1 RNA extraction, amplification, and sequencing

HIV-1 RNA was extracted using the MagNA Pure LC Total Nucleic Acid Isolation Kit and the MagNA Pure LC 2.0 instrument (Roche, Germany) for automated extraction. The extracted RNA was subjected to nested polymerase chain reaction (PCR) with the One Step PrimeScript RT-PCR Kit (Takara, Dalian, China) to amplify *pol* gene fragments (HXB2: 2253-3464). PCR-positive amplification products were purified and sequenced. The chromatogram data were cleaned and assembled using Sequencher v5.4.6 (Gene Codes, Ann Arbor, MI). Only sequences over 900 nucleotides were retained for analysis, as shorter sequences may reduce the accuracy of network inference (14).

### 2.3 HIV-1 subtype analysis

The sequences with mixed base proportions greater than 5% were excluded using the Quality Control online tool in the Los Alamos National Laboratory HIV database (https://www.hiv.lanl.gov). Major internationally prevalent strains (subtypes A-D) and the main CRFs in China were downloaded from the HIV database as reference sequences. The obtained sequences were aligned with the reference sequences using MAFFT (version 7.526), followed by manual editing using BioEdit (version 7.0.5.3). The maximum likelihood (ML) phylogenetic tree was constructed using FastTree (version 2.1.11), with the nucleotide substitution model set to GTR+Gamma (15). The resulting ML phylogenetic tree was visualized using the online tool iTOL v7 (https://itol.embl.de/). Sequences clustering with reference sequences and showing a bootstrap value ≧ 70% were identified as the same subtype (16). For sequences whose subtypes could not be determined, the HIV BLAST online tool was used for further identification. Sequences that could not be assigned a subtype after two rounds of analysis were defined as unique recombinant forms (URFs).

### 2.4 Drug resistance analysis

The cleaned *pol* gene sequences were uploaded to the Stanford University HIV Drug Resistance Database (https://hivdb.stanford.edu) for drug resistance analysis, to identify resistance-related mutations and assess drug resistance to protease inhibitors (PIs), nucleoside reverse transcriptase inhibitors (NRTIs), and non-nucleoside reverse transcriptase inhibitors (NNRTIs). Drug resistance was classified into five levels based on the scoring system provided by the database: susceptible (S, 0–9 points), potential low-level resistance (PLR, 10–14 points), low-level resistance (LR, 15–29 points), intermediate-level resistance (IR, 30–59 points), and high-level resistance (HR, ≥60 points). Low-level resistance or above was designated as having drug resistance.

### 2.5 Molecular transmission network construction and analysis

Genetic distances between sequences were calculated using HYPHY (version 2.2.4) based on the pairwise Tamura-Nei 93 model. According to Technical Guidelines for Monitoring and Intervention of HIV Transmission Network (2021 Trial Edition) issued by the China CDC, a genetic distance threshold of 0.005 was applied for HIV-1 subtypes. Molecular transmission networks were constructed and visualized using Cytoscape (version 3.8.0) (17).

After constructing the network, the Network Analyzer tool in Cytoscape was used to analyze network characteristics, including the number of sequences (nodes), the number of connections (edges), and the degree of each node (i.e., the number of connections with other individuals). To identify transmission clusters with active expansion, the number of newly added nodes in the molecular transmission network was tracked annually. Transmission clusters were categorized as follows: Sustained growth clusters: Clusters that added at least one new node each year after formation. Newly formed clusters: Clusters that first formed in 2024. Growth-reactivated clusters: Clusters that added at least one new node in 2024 but had no growth in 2023 or 2022-2023. No recent growth clusters: Clusters with no new nodes added in 2024 (18).

Each node was matched with its corresponding reporting region. Connections between nodes within the same region were defined as intra-regional transmission, while those between different regions were classified as inter-regional transmission. Based on this, heatmaps of transmission intensity matrices for different HIV-1 subtypes were generated using Python (version 3.8.2). In addition, for large transmission clusters (≥5 nodes) identified in the molecular transmission network, we further analyzed inter-regional transmission patterns and identified key transmission nodes within these clusters. These findings provided a foundation for subsequent epidemiological investigations and the development of targeted intervention strategies.

### 2.6 Statistical analysis

Continuous and discrete variables were categorized according to clinically meaningful or literature-derived cut-off values to facilitate statistical analysis. All sociodemographic and clinical data were obtained from the China HIV/AIDS Prevention and Control Information System. The dataset was complete, and no missing data were observed. Statistical comparisons were performed using Fisher’s exact test and Chi-square tests. Fisher’s exact test was applied when at least one expected frequency was less than 1. The Cochran-Armitage test was used to assess statistically significant trends in increasing or decreasing proportions over the years or across age groups. Logistic regression analysis was used to identify factors associated with clustering rates in the molecular transmission network. Variables examined included demographic characteristics (e.g., age, gender, occupation), sociobehavioral factors (e.g., transmission route, number of sexual partners), and biological indicators (e.g., CD4^+^ cell count). Univariate logistic regression was initially conducted, and variables with the *p* < 0.10 were subsequently included in the multivariate logistic regression model. All statistical tests were two-sided, and *p* < 0.05 was considered statistically significant. Data analysis was conducted using SPSS (version 26.0).

## 3 Results

### 3.1 Sociodemographic characteristics

A total of 1,440 newly reported HIV-1 infections from Nantong were collected from January 2021 to December 2024. The *pol* gene sequences of 1,303 samples were successfully amplified and sequenced. Among them, 11 sequences were excluded due to poor sequencing quality, and 89 were excluded due to the inability to match the demographic information. Ultimately, 1,203 subjects were retained for subsequent analysis.

**Figure 1:**
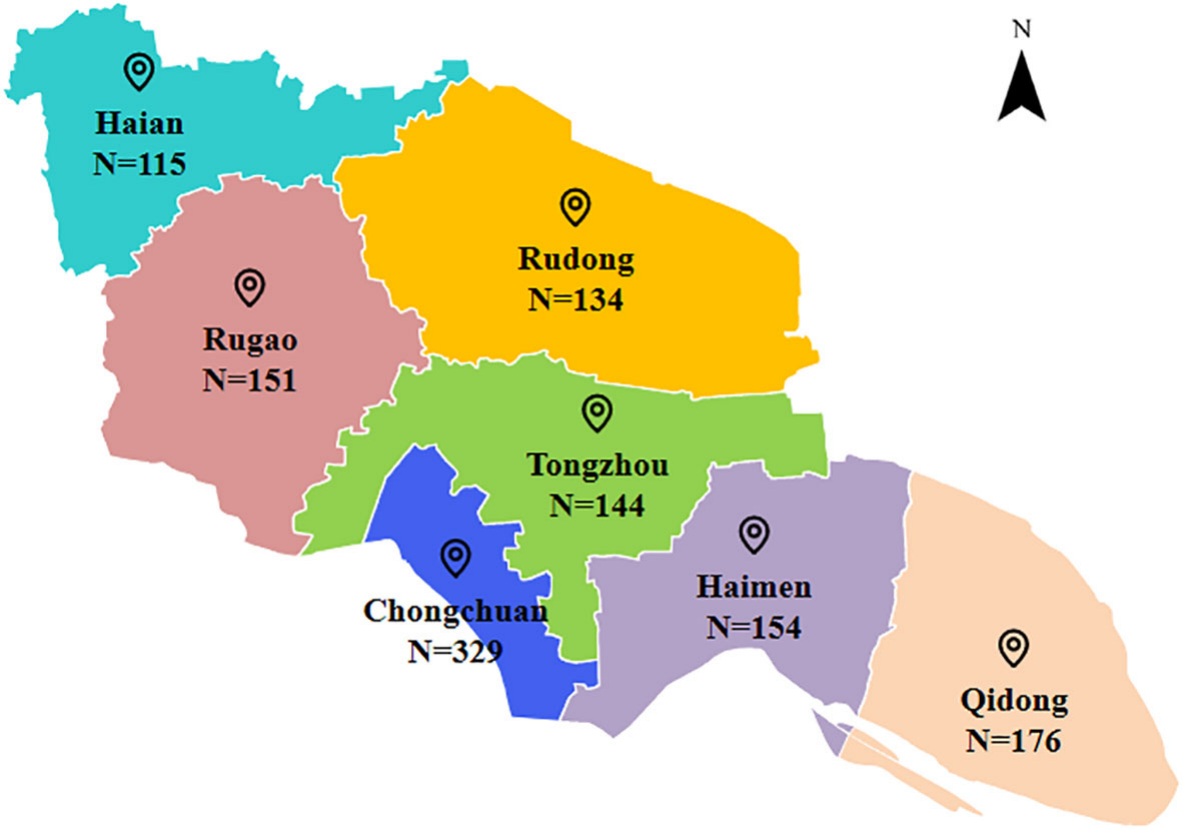
Regional distribution of 1,203 newly reported HIV-1 in Nantong, 2021-2024. Administrative divisions of Nantong City and the reported number of HIV-1 subjects in different regions.

Among the 1,203 subjects, the male-to-female ratio was 4.3:1. The mean age was 46.4 ± 15.6 years, with a median age of 49 years (IQR: 33-58), while aged 50 years or older accounted for 49.0%. Married individuals accounted for 52.8%. Most subjects (60.4%) had a junior high school or below education, while farmers represented 33.4%. Regarding transmission routes, 55.7% of subjects were infected through homosexual contact, while 43.9% were infected through heterosexual contact. Two individuals were infected through injection drug use. Both reported sharing syringes with others in entertainment venues. Additionally, 14.1% of the subjects had a self-reported history of sexually transmitted diseases (STDs). The median number of non-marital sexual partners was 3 (IQR: 2-5). The median baseline CD4^+^ cell count was 252 cells/μL (IQR: 196-354). Geographically, the region with the largest number of newly reported HIV-1 individuals was Chongchuan, accounting for 27.3% (**Figure 1**).

Statistical analysis showed significant differences in occupation, history of STDs, number of non-marital sexual partners, baseline CD4^+^ cell count, and regional distribution among newly reported HIV-1 subjects in different years (*p* < 0.05) (**Table 1**).

**Table 1:** Sociodemographic characteristics of 1203 newly reported HIV-1 subjects in Nantong, 2021-2024.

Variable	Number(%) N=1203	2021 n=344	2022 n=232	2023 n=317	2024 n=310	χ^2^	*P*
Age						9.779	0.369
<25	116 (9.6)	34 (9.9)	29 (12.5)	26 (8.2)	27 (8.7)		
25-	498 (41.4)	137 (39.8)	99 (42.7)	135 (42.6)	127 (41.0)		
50-	522 (43.4)	152 (44.2)	86 (37.1)	143 (45.1)	141 (45.5)		
≧70	67 (5.6)	21 (6.1)	18 (7.8)	13 (4.1)	15 (4.8)		
Gender						0.339	0.953
Male	977 (81.2)	276 (80.2)	189 (81.5)	258 (81.4)	254 (81.9)		
Female	226 (18.8)	68 (19.8)	43 (18.5)	59 (18.6)	56 (18.1)		
Ethnicity		1.961	0.581
Han	1168 (97.1)	337 (98.0)	225 (97.0)	308 (97.2)	298 (96.1)		
Others	35 (2.9)	7 (2.0)	7 (3.0)	9 (2.8)	12 (3.9)		
Birthplace						4.681	0.197
Nantong	916 (76.1)	263 (76.5)	184 (79.3)	240 (75.7)	229 (73.9)		
Others	287 (23.9)	81 (23.5)	48 (20.7)	77 (24.3)	91 (29.4)		
Marital status						6.593	0.360
Married	635 (52.8)	174 (50.6)	122 (52.6)	162 (51.1)	177 (57.1)		
Unmarried	305 (25.4)	83 (24.1)	62 (26.7)	82 (25.9)	78 (25.2)		
Divorced/Widowed	263 (21.9)	87 (25.3)	48 (20.7)	73 (23.0)	55 (17.7)		
Education						12.943	0.165
Primary school or below	270 (22.4)	79 (23.0)	52 (22.4)	69 (21.8)	70 (22.6)		
Junior high school	457 (38.0)	130 (37.8)	88 (37.9)	122 (38.5)	117 (37.7)		
High school	241 (20.0)	68 (19.8)	50 (21.6)	57 (18.0)	66 (21.3)		
Junior college or above	235 (19.5)	67 (19.5)	42 (18.1)	69 (21.8)	57 (18.4)		
Occupation						41.294	<0.001
Farmer	402 (33.4)	120 (34.9)	90 (38.8)	100 (31.5)	92 (29.7)		
Worker	217 (18.0)	43 (12.5)	40 (17.2)	70 (22.1)	64 (20.6)		
Unemployed	196 (16.3)	54 (15.7)	33 (14.2)	48 (15.1)	61 (19.7)		
Business services	168 (14.0)	71 (20.6)	31 (13.4)	39 (12.3)	27 (8.7)		
Retired	54 (4.5)	10 (2.9)	10 (4.3)	15 (4.7)	19 (6.1)		
Student	40 (3.3)	13 (3.8)	8 (3.4)	8 (2.5)	11 (3.5)		
Other and unknown	126 (10.5)	33 (9.6)	20 (8.6)	37 (11.7)	36 (11.6)		
Transmission route						3.517	0.742^a^
Homosexual	670 (55.7)	193 (56.1)	135 (58.1)	171 (53.9)	171 (55.2)		
Heterosexual	528 (43.9)	149 (43.3)	97 (41.8)	145 (45.7)	137 (44.2)		
Others	5 (0.4)	2 (0.6)	0 (0)	1 (0.3)	2 (0.6)		
History of STDs^c^						30.586	<0.001
No	972 (80.8)	275 (80.0)	196 (84.5)	273 (86.1)	228 (73.5)		
Yes	170 (14.1)	47 (13.7)	30 (12.9)	40 (12.6)	53 (17.1)		
Unknown	61 (5.1)	22 (6.4)	6 (2.6)	4 (1.3)	29 (9.4)		
Number of non-marital sexual partners^b^						27.669	<0.001
1-	633 (66.8)	176 (55.5)	118 (55.7)	158 (55.4)	181 (62.8)		
4-	367 (24.0)	96 (30.3)	67 (31.6)	107 (37.5)	97 (33.7)		
≧10	102 (9.2)	45 (14.2)	27 (12.7)	20 (7.0)	10 (3.5)		
Baseline CD4^+^ cell count (cells/μL)							
<200	295 (24.5)	103 (29.9)	60 (25.9)	49 (15.5)	83 (26.8)	28.990	<0.001
200-	754 (62.7)	187 (54.3)	146 (62.9)	232 (73.2)	189 (61.0)		
≧500	104 (8.6)	35 (10.2)	17 (7.3)	26 (8.2)	26 (8.4)		
Unknown	50 (4.2)	19 (5.5)	9 (3.9)	10 (3.2)	12 (3.9)		
Reported region						65.081	<0.001
Chongchuan	329 (27.3)	75 (21.8)	35 (15.1)	102 (32.2)	117 (37.7)		
Qidong	176 (14.6)	54 (15.7)	41 (17.7)	47 (14.8)	34 (11.0)		
Haimen	154 (12.8)	48 (14.0)	39 (16.8)	37 (11.7)	30 (9.7)		
Rugao	151 (12.6)	38 (11.0)	39 (16.8)	36 (11.4)	38 (12.3)		
Tongzhou	144 (12.0)	56 (16.3)	31 (13.4)	34 (10.7)	23 (7.4)		
Rudong	134 (11.1)	41 (11.9)	31 (13.4)	33 (10.4)	29 (9.4)		
Haian	115 (9.6)	32 (9.3)	16 (6.9)	28 (8.9)	39 (12.6)		

a : Fisher’s exact test was used to verify.

b : A total of 101 individuals were excluded from the analysis due to the absence of non-marital sexual partners.

c : STDs, sexually transmitted diseases.

### 3.2 HIV-1 subtype distribution

**Figure 2:**
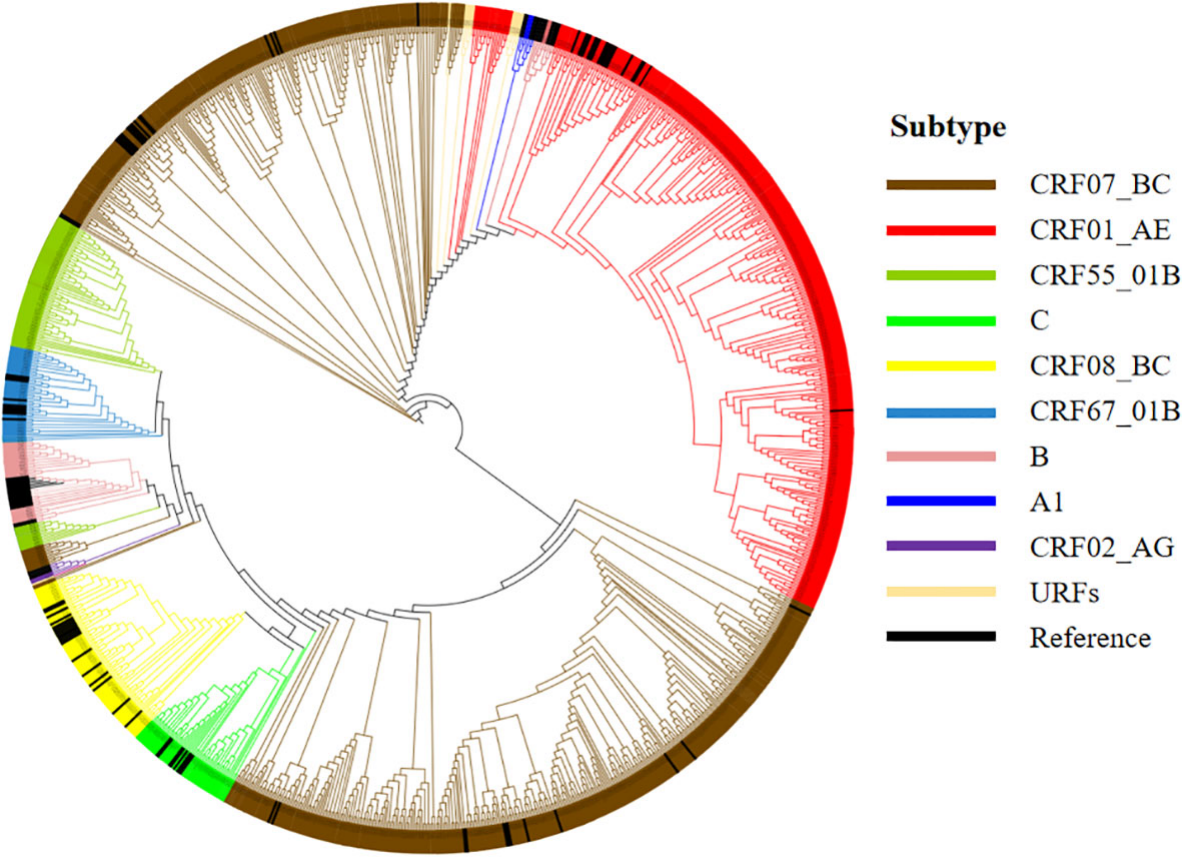
Phylogenetic tree analysis of pol gene nucleotide sequences from 1,203 newly reported HIV-1 subjects. Sequences that cluster with reference strains and show a bootstrap value ≥70% are assigned to the same subtype. URFs, unique recombinant forms.

Among the 1,203 successfully amplified sequences, the most prevalent CRF was CRF07_BC (48.7%), followed by CRF01_AE (29.5%), CRF55_01B (6.6%), C (4.8%), CRF08_BC (4.6%), CRF67_01B (2.7%), B (1.7%), A1 (0.1%), and CRF02_AG (0.1%). Additionally, 14 sequences (1.2%) did not cluster with any known reference sequences and were classified as URFs (**Figure 2**).

**Figure 3:**
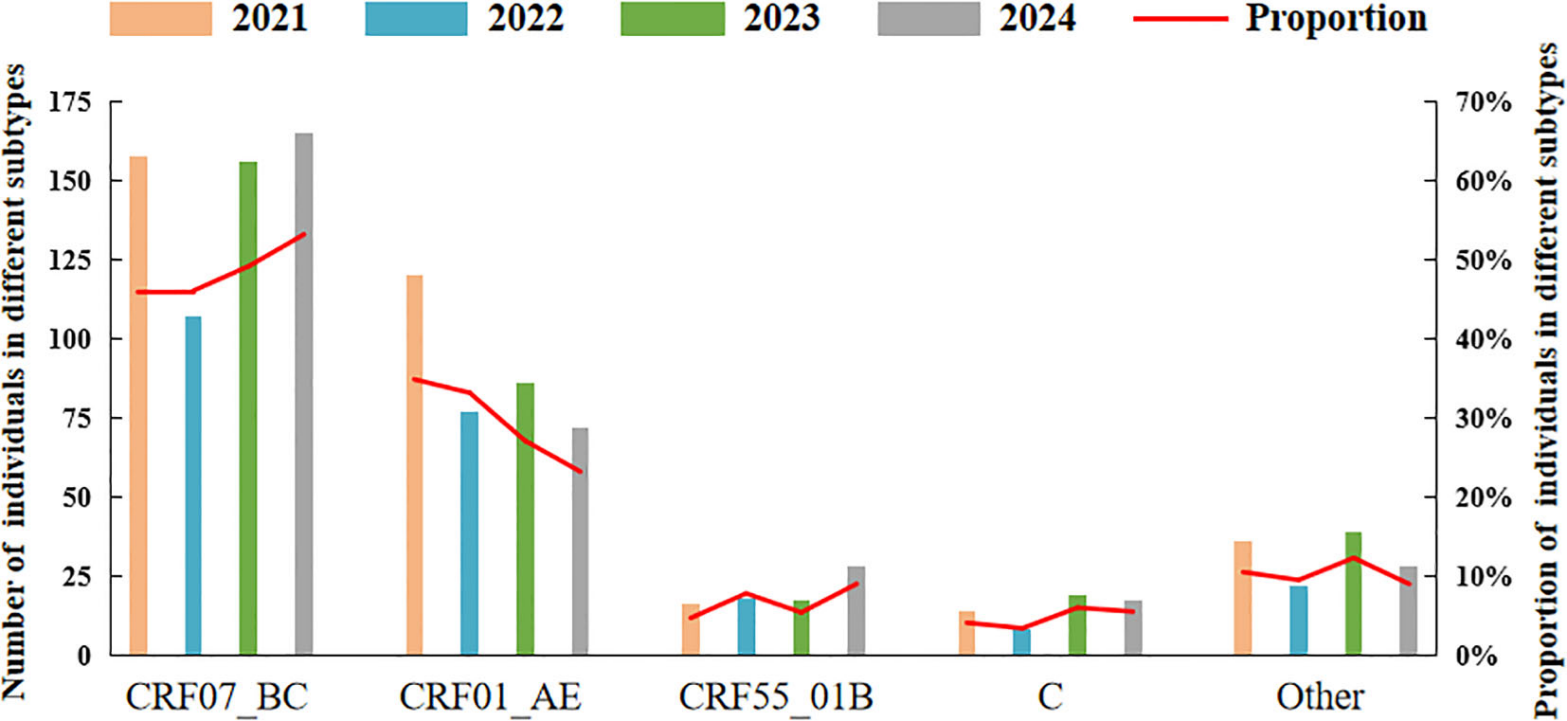
Distribution of HIV-1 subtypes among 1203 newly reported HIV-1 subjects. The bars represent the number of individuals for each HIV-1 subtype, while the red line indicates the proportion of each subtype over the four years.

CRF07_BC was the predominant subtype. The proportion of CRF07_BC increased over the period from 2021 to 2024 (trend χ²=3.881, p=0.043), with the proportions of 45.9% (158/344), 46.1% (107/232), 49.2% (156/317), and 53.2% (166/310), respectively. In contrast, the proportion of CRF01_AE showed a decreased trend (trend χ²=12.612, p<0.001), with the proportions of 34.9% (120/344), 33.2% (77/232), 27.1% (86/317), and 23.2% (72/310) from 2021 to 2024. Meanwhile, the changes in the proportions of CRF55_01, C, and other subtypes were not statistically significant (trend χ²=3.301, 1.467, 0.460, respectively, p>0.05) (**Figure 3**).

### 3.3 ART pretreatment HIV-1 drug resistance

**Figure 4:**
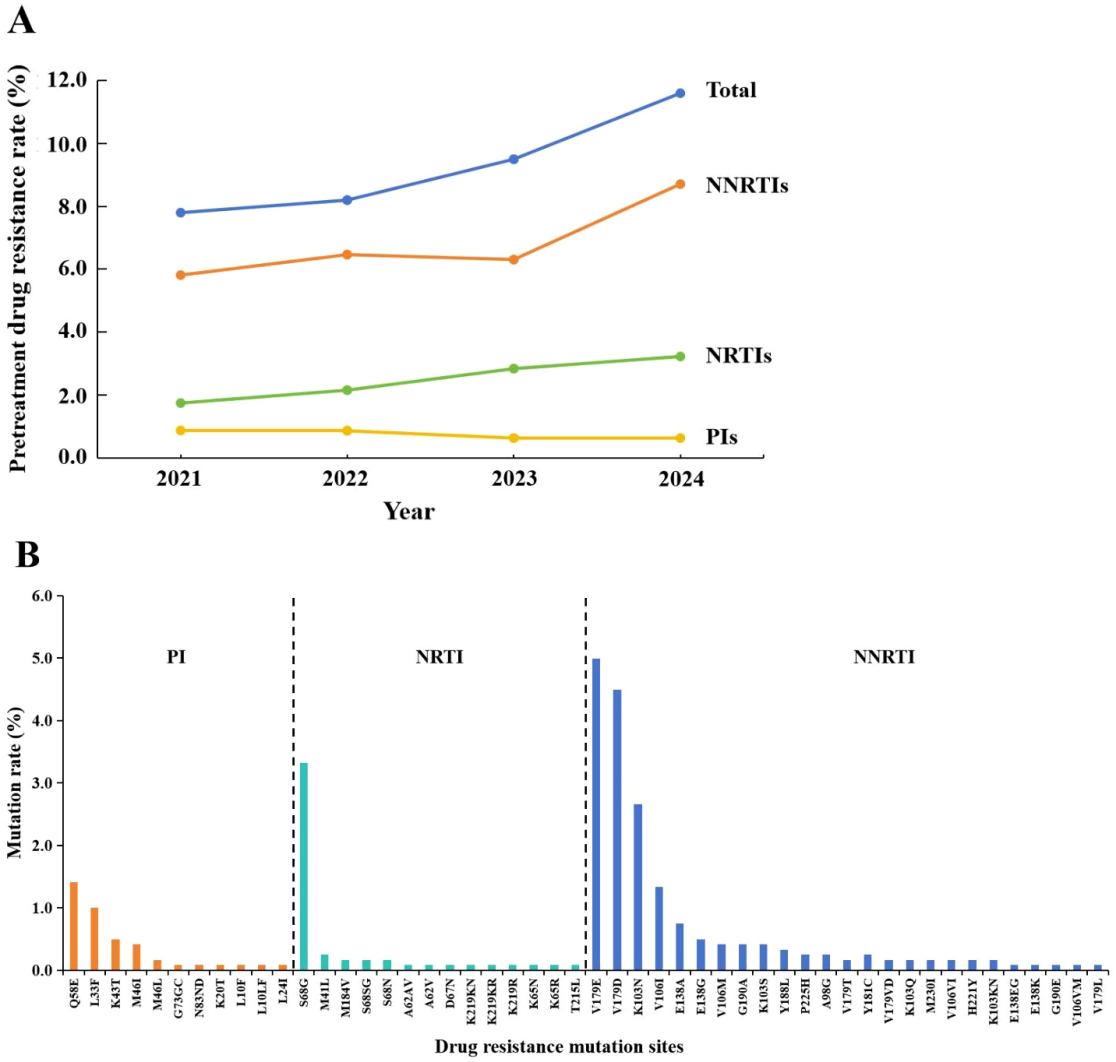
Pretreatment drug resistance rates and frequency of drug resistance-associated mutations among 1203 newly reported HIV-1 subjects. **(A)** Pretreatment drug resistance rates for the total population and by drug class. **(B)** Frequency of drug resistance-associated mutations for each drug class. NNRTIs, non-nucleoside reverse transcriptase inhibitors; NRTIs, nucleoside reverse transcriptase inhibitors; PIs, protease inhibitors.

The overall PDR rate among the subjects was 9.3% (112/1,203). The rates from 2021 to 2024 were 7.8%, 8.2%, 9.5%, and 11.6%, respectively(trend χ²=2.881, *p* = 0.090). Resistance to NNRTIs was the most prevalent form, with the rate rising from 5.81% in 2021 to 8.71% in 2024 (trend χ²=1.803, *p* = 0.179). A gradual upward trend was also observed in the resistance rate to NRTIs (trend χ²=1.723, *p* = 0.189). In contrast, resistance rates to PIs remained relatively stable throughout the study period (trend χ²=0.173, *p* = 0.678) (**Figure 4A**).

Among the 112 drug resistant individuals, 82 (6.8%) exhibited resistance to NNRTIs, 30 (2.5%) to PIs, and 9 (0.7%) to NRTIs. Co-resistance was observed in 6 individuals for both PIs and NNRTIs, and in 3 for both NNRTIs and NRTIs. No individuals resistant to all three drug classes were identified.

A total of 50 drug resistance-associated mutations were identified, including 11 associated with PIs, 14 with NRTIs, and 25 with NNRTIs. Among NNRTI-related mutation sites, the most common was V179E (5.0%), followed by V179D (4.5%), K103N (2.7%), and V106I (1.3%). For NRTI-related mutations, the most common was S68G (3.3%), with M41L and M184V each detected in 0.2% of subjects. Among PI-related mutations, Q58E was the most prevalent (1.4%), followed by L33F (1.0%) and K43T (0.4%) (**Figure 4B**).

### 3.4 Molecular transmission network analysis of 1,203 newly reported HIV-1 subjects

The molecular transmission network was constructed using a 0.5% genetic distance threshold and visualized for analysis. Among the 1,203 valid HIV-1 sequences, 326 (27.1%) were incorporated into the network. A total of 116 transmission clusters were identified, with cluster sizes ranging from 2 to 17 sequences. Of these, 31 clusters (26.7%) consisted of three or more sequences, while the remaining 85 clusters contained only two.

**Figure 5:**
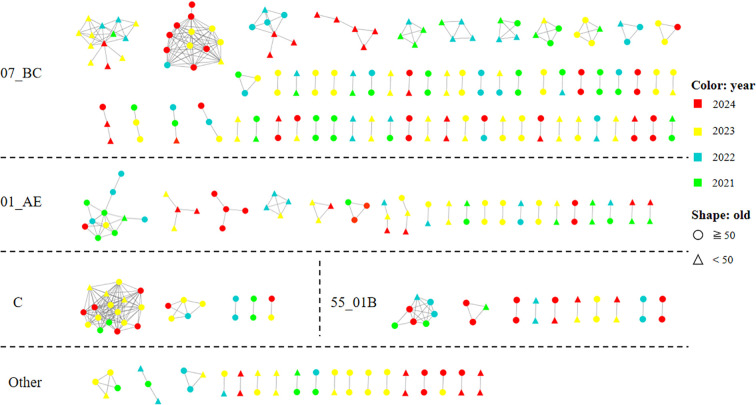
The molecular transmission network of 1203 newly reported HIV-1 subjects. Each row represents different HIV-1 subtypes, with the nodes indicating individuals or sequences. Each color corresponds to a specific year. Nodes with circles represent individuals aged 50 years or older, while triangles represent individuals aged under 50 years.

**Figure 6:**
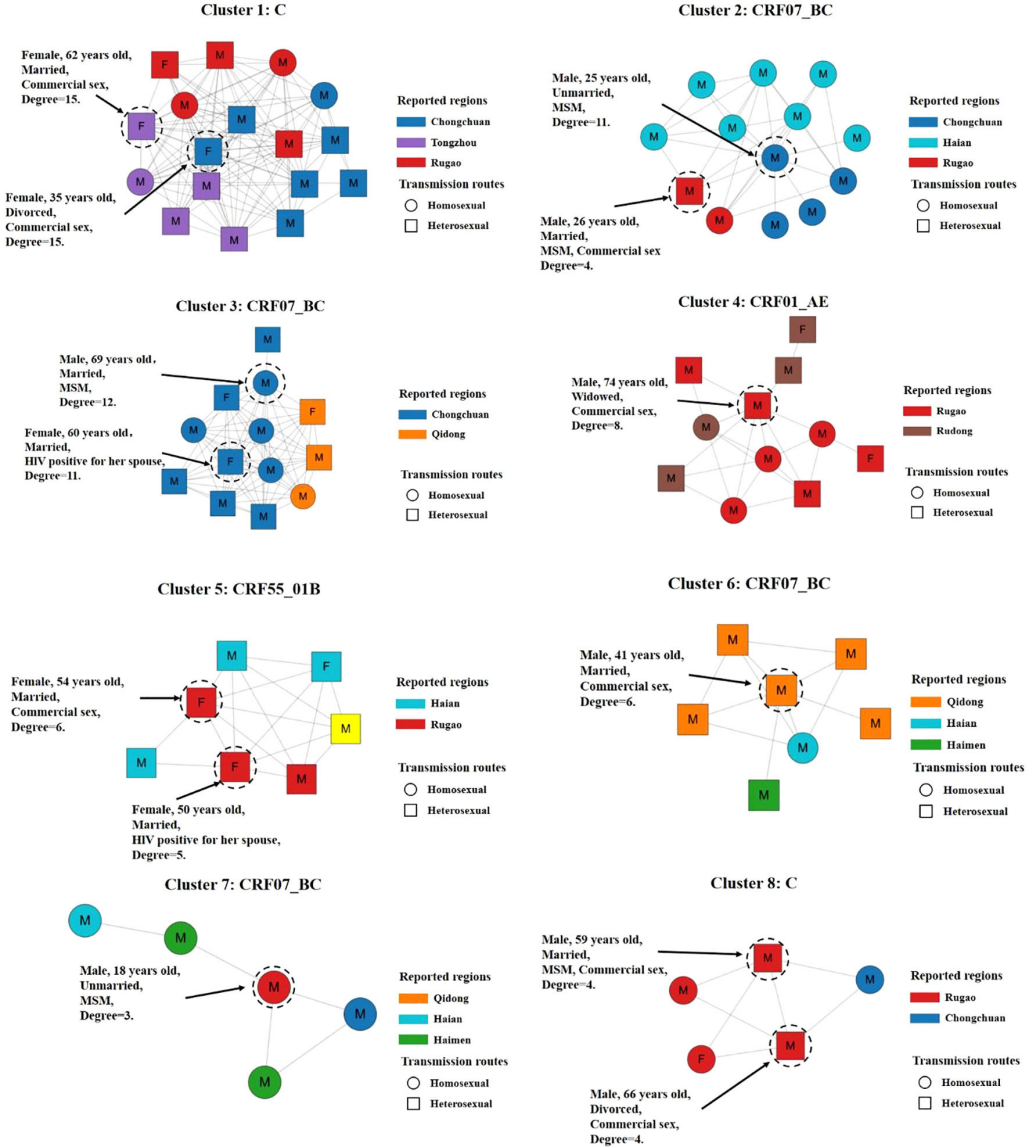
Cross-regional spread of large transmission clusters (≥5 nodes) among 1203 newly reported HIV-1 subjects in Nantong. Colors indicate different reported regions. Shapes indicate different transmission routes. Individuals at high risk of HIV transmission are circled with black dashed lines and indicated by black arrows.

When categorized by HIV-1 subtype, the molecular transmission network included 62 CRF07_BC clusters, 21 CRF01_AE clusters, 10 CRF55_01B clusters, 5 C clusters, and 18 clusters consisting of other subtypes. Subjects aged 50 years or older accounted for 55.8% (182/326) of the networked subjects and were distributed across 85 clusters. The largest cluster was associated with C and included 17 sequences, primarily representing older individuals with histories of commercial heterosexual sex. The second cluster belonged to CRF07_BC, comprising 13 sequences predominantly involving young men who engaged in homosexual contact. The third cluster was also CRF07_BC, containing 13 sequences, mostly from older individuals who engaged in both commercial heterosexual sex and homosexual behavior (**Figures 5**, **6**).

To identify clusters with sustained expansion, we examined the yearly addition of new nodes within each transmission cluster. The results showed that 16 clusters exhibited a sustained growth trend. In 2024, 19 new clusters were formed, and 10 clusters showed “growth reactivation” characteristics. No new nodes were observed in 71 clusters (**Figure 5**).

Univariate analysis found that variables of age, education, occupation, transmission route, time of diagnosis, viral subtype, and baseline CD4^+^ cell count were significantly associated with being within the molecular transmission network (*p* < 0.10). These variables were entered into the multivariate logistic regression model, which showed that individuals aged 70 years or older had a significantly higher likelihood of being within the molecular transmission network compared to those aged under 25 years (OR = 2.17, 95% CI:1.02-4.62), with a positive trend observed across increasing age groups (trend χ²=9.695, p=0.002). Compared to individuals diagnosed in 2021, those diagnosed in 2022 (OR = 1.62, 95%CI:1.06-2.47), 2023 (OR = 2.37, 95%CI:1.62-3.48), and 2024 (OR = 1.77, 95%CI:1.19-2.63) were significantly more likely to be within the network. CD4^+^ cell counts were also associated with clustering: individuals with counts of 200–500 cells/μL (OR = 1.85, 95%CI:1.29-2.65) and >500 cells/μL (OR = 1.87, 95%CI:1.08-3.27) had higher clustering probabilities compared to those with counts <200 cells/μL. With respect to HIV-1 subtypes, individuals infected with CRF01_AE were less likely to cluster compared to those with CRF07_BC (OR = 0.58, 95%CI:0.41-0.82), whereas individuals infected with subtype C demonstrated a significantly higher clustering rate (OR = 1.98, 95%CI:1.11-3.54) (**Table 2**).

**Table 2:** Analysis of factors associated with being within the molecular transmission networks.

Variables	Cases n=1203	Cluster (%) n=326	Univariate logistic analysis		Multivariate logistic analysis	
			OR (95%CI)	*P*	OR (95%CI)	*P*
Age						
<25	116	27 (23.3)	1.00		1.00	
25-	498	117 (23.9)	0.97 (0.60-1.56)	0.892	1.03 (0.62-1.69)	0.912
50-	522	155 (29.7)	1.44 (0.90-2.31)	0.125	1.46 (0.85-2.53)	0.173
≧70	67	27 (40.3)	2.23 (1.16-4.27)	0.016	2.17 (1.02-4.62)	0.044
Gender						
Male	977	259 (26.5)	1.00			
Female	226	67 (29.6)	1.17 (0.85-1.61)	0.339		
Ethnicity						
Han	1168	320 (27.4)	1.00			
Others	35	6 (17.1)	0.55 (0.23-1.33)	0.185		
Birthplace						
Nantong	916	254 (27.7)	1.00			
Others	287	72 (25.1)	0.79 (0.58-1.08)	0.137		
Marital status						
Married	635	177 (27.9)	1.00			
Unmarried	305	77 (25.2)	0.87 (0.64-1.19)	0.396		
Divorced/Widowed	263	72 (27.4)	0.98 (0.71-1.35)	0.880		
Education						
Primary school or below	270	90 (33.3)	1.00			
Junior high school	457	122 (26.7)	0.60 (0.32-1.13)	0.116	0.62 (0.31-1.23)	0.171
High school	241	56 (23.2)	0.50 (0.25-0.98)	0.043	0.58 (0.27-1.24)	0.158
Junior college or above	235	58 (24.7)	0.54 (0.28-1.06)	0.072	0.68 (0.30-1.51)	0.339
Occupation						
Farmer	402	113 (28.1)	1.00			
Worker	217	46 (21.2)	0.69 (0.47-1.02)	0.061	0.89 (0.57-1.39)	0.614
Unemployed	196	46 (23.5)	0.78 (0.53-1.16)	0.229	0.95 (0.62-1.47)	0.818
Business services	168	44 (26.2)	0.91 (0.60-1.36)	0.640	1.49 (0.92-2.42)	0.104
Retired	54	25 (46.3)	2.33 (1.24-4.69)	0.008	1.74 (0.88-3.42)	0.109
Student	40	8 (20.0)	0.64 (0.29-1.43)	0.276	1.06 (0.38-2.98)	0.913
Other and unknown	126	44 (34.9)	1.37 (0.90-2.10)	0.145	1.80 (1.09-2.95)	0.021
Transmission route						
Homosexual	670	165 (24.6)	1.00			
Heterosexual	528	161 (30.5)	1.34 (1.04-1.73)	0.024	1.16 (0.86-1.56)	0.330
Others	5	0 (0)	–			
History of STDs^b^						
No	972	267 (27.5)	1.00			
Yes	170	45 (26.5)	0.95 (0.66-1.37)	0.788		
Unknown	61	14 (23.0)	0.79 (0.43-1.45)	0.443		
Number of non-marital sexual partners^a^						
1-	633	162 (25.6)				
4-	367	98 (26.7)	1.06 (0.79-1.42)	0.700		
≧10	102	28 (27.5)	1.10 (0.69-1.76)	0.691		
Year of diagnosis						
2021	344	63 (18.3)	1.00			
2022	232	59 (25.4)	1.52 (1.02-2.27)	0.041	1.62 (1.06-2.47)	0.027
2023	317	115 (36.3)	2.52 (1.76-3.60)	0.000	2.37 (1.62-3.48)	<0.001
2024	310	89 (28.7)	1.80 (1.24-2.59)	0.002	1.77 (1.19-2.63)	0.005
Subtype						
07_BC	586	171 (29.2)	1.00		1.00	
01_AE	355	61 (17.2)	0.51 (0.36-0.70)	<0.000	0.53 (0.38-0.74)	<0.001
55_01B	79	26 (32.9)	1.20 (0.72-1.98)	0.482	1.32 (0.78-2.23)	0.300
C	58	28 (48.3)	2.13 (1.23-3.67)	0.007	1.98 (1.11-3.54)	0.021
Others	125	40 (32.0)	1.19 (0.79-1.80)	0.407	1.34 (0.87-2.08)	0.186
Drug-resistant						
Yes	112	24 (21.4)	1.00			
No	1091	302 (27.7)	1.40 (0.88-2.25)	0.158		
Baseline CD4^+^ cell count (cells/μL)						
<200	295	51 (17.3)	1.00			
200-500	754	235 (31.2)	2.17 (1.54-3.04)	<0.001	1.85 (1.29-2.65)	<0.001
>500	104	31 (29.8)	2.03 (1.21-3.41)	0.007	1.87 (1.08-3.27)	0.027
Unknown	50	9 (18.0)	1.05 (0.48-2.30)	0.902	1.03 (0.46-2.32)	0.943
Reported region						
Chongchuan	329	89 (27.1)	1.00			
Qidong	176	51 (29.0)	1.10 (0.73-1.65)	0.645		
Haimen	154	39 (25.3)	0.91 (0.59-1.42)	0.689		
Rugao	151	46 (30.5)	1.18 (0.77-1.80)	0.440		
Tongzhou	144	33 (22.9)	0.80 (0.51-1.27)	0.345		
Rudong	134	37 (27.6)	1.03 (0.66-1.61)	0.902		
Haian	115	31 (27.0)	1.00 (0.62-1.61)	0.984		

a A total of 101 individuals were excluded from the analysis due to the absence of non-marital sexual partners.

b STDs, sexually transmitted diseases.

### 3.5 Regional transmission patterns of the subjects

**Figure 7:**
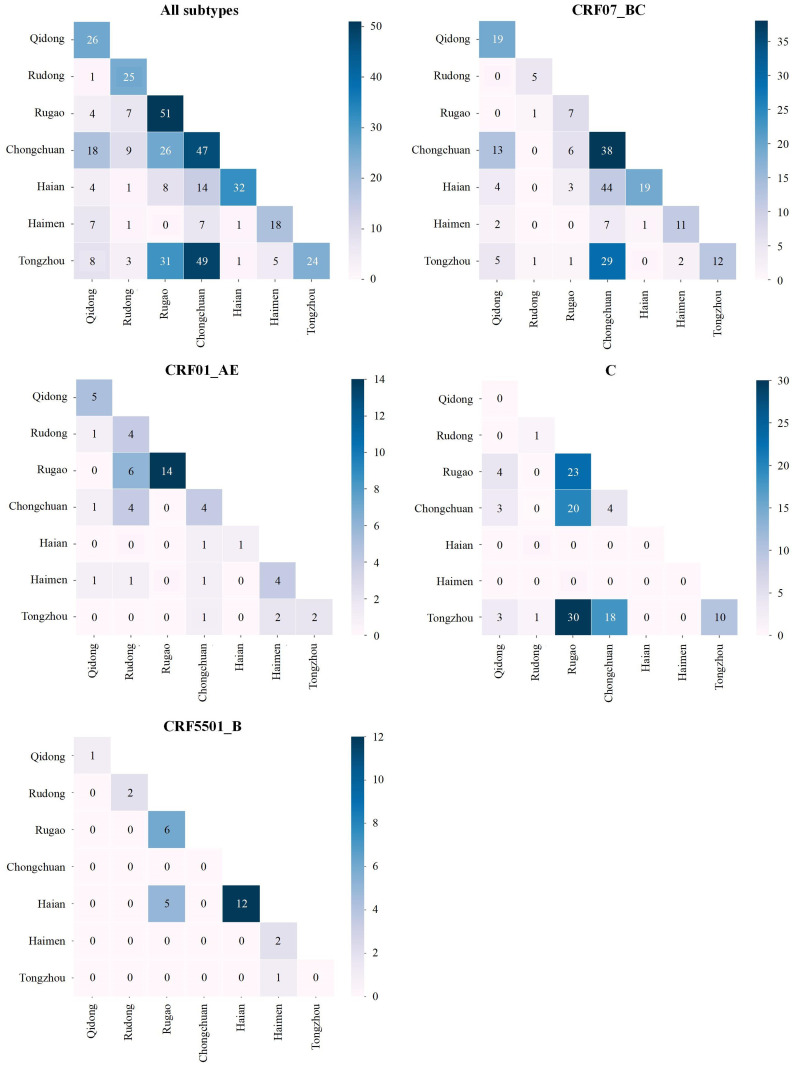
Intensity matrices of HIV-1 transmission links between cities in Nantong, 2021-2024. The color of the grid cell at the intersection of two cities represents the number of linkages between the HIV-1 individuals in two cities.

Based on molecular transmission network analysis constructed from HIV-1 *pol* gene sequences and associated demographic data, the strength of intra-city and inter-city transmission links was inferred by identifying genetic clustering among subjects from different regional locations. Overall, 52.1% of transmission events occurred between newly reported individuals residing in the same city. Among the subtypes, the proportions of intra-city transmission were 57.5% for CRF07_BC, 64.1% for CRF01_AE, 32.5% for subtype C, and 79.3% for CRF55_01B. At the regional level, the proportion of inter-city transmission was lowest in Rudong (46.8%) and highest in Tongzhou (80.2%) (**Figure 7**).

Further analysis of inter-regional transmission within large transmission clusters (nodes ≥5) (19) revealed subtype-specific patterns of inter-city spread among newly reported individuals. For the CRF07_BC, transmission between Chongchuan and Qidong was primarily associated with commercial heterosexual sex, whereas transmission between Chongchuan and Haian was mainly linked to male-to-male sexual contact. For the CRF01_AE, inter-city transmission predominantly occurred between Rugao and Rudong, involving both commercial and male-to-male sexual behaviors. Transmission of subtype C was relatively localized, with two key nodes, both female sex workers, serving as central hubs in the inter-regional network, primarily linking Chongchuan, Tongzhou, and Rugao. The CRF55_01B was mainly transmitted through commercial sex between Haian and Rugao (**Figure 6**).

## 4 Discussion

Similar to other regions in China (20, 21), newly diagnosed HIV-1 in Nantong between 2021 and 2024 predominantly involved males (81.2%), with 99.6% of subjects were attributed to sexual transmission. The median number of non-marital sexual partners was 3 (IQR: 2-5). The history of STDs was higher than that in the general population (22). These findings highlighted the critical role of high-risk sexual behaviors in driving the local AIDS epidemic. We found that the proportion of HIV-1 transmission among young people under 25 years of age and student populations remained stable. This could be attributed to the widespread implementation of AIDS prevention campaigns and health education programs in school settings (23). Moreover, 71.1% of the subjects had CD4^+^ cell counts below 350 cells/μL at the time of initial diagnosis, indicating that immune function may have been compromised. This suggested a delay in the detection of HIV infection.

Our study found that the predominant HIV-1 subtypes in Nantong were CRF07_BC and CRF01_AE, which is consistent with findings from molecular epidemiological studies conducted in Nanjing Zhejiang Province, and 30 other provinces across China (24–26). In the past two decades, the distribution of HIV-1 genotypes in Jiangsu Province has undergone significant changes. In 2000-2001, the dominant subtypes were subtype C and B’; however, in recent years, they have been gradually replaced by CRF01_AE and CRF07_BC, along with an increasing presence of URFs (27, 28). During the study period, the proportion of CRF01_AE infections exhibited a downward trend, while the proportion of CRF07_BC infections continued to rise. Previous studies suggested that CRF07_BC originated among people who inject drugs and subsequently spread to MSM and heterosexual individuals, thereby facilitating its widespread transmission and increasing prevalence over time (29–31). The declining proportion of CRF01_AE infections may be related to the faster decline in CD4^+^ cell counts and earlier disease progression, which can lead to earlier onset of symptoms and reduce the window of opportunity for transmission (32).

In our study, the overall PDR rate among HIV-1 infections in Nantong was 9.3%. According to the World Health Organization classification criteria (33), this placed Nantong within the moderate PDR prevalence category, higher than those in neighboring Shanghai (4.8%) (34) and the national average in China (7.8%) (35). From 2021 to 2024, the local PDR rate increased from 7.8% to 11.6%, indicating an upward trend and suggesting a growing threat of PDR. The frequently observed resistance-associated mutations in our study were K103N, V179E, V179D, V106I, S68G, and Q58E. Among these, K103N was the most commonly transmitted resistance mutation, conferring high-level resistance to nevirapine (NVP) and efavirenz (EFV), though without reducing susceptibility to rilpivirine (RPV), etravirine (ETR), or doravirine (DOR) (36). V179E and V179D are associated with potential low-level resistance to NNRTIs (37); however, when V179D co-occurs with V106I, the virus exhibits significant resistance to NNRTIs such as EFV and NVP (38). S68G is a polymorphic mutation selected by tenofovir (TDF), but its impact on NRTI susceptibility remains uncertain (39). Q58E is the most common PI associated resistance mutation, conferring low-level resistance to tipranavir (TPV) (40). Currently, lamivudine (3TC), EFV, and NVP are widely used as part of China’s first-line ART regimens and are provided free of charge (41). Notably, these drugs were also associated with the highest rates of resistance in this study. Prolonged reliance on a limited range of ART medications may facilitate the emergence and transmission of drug-resistant HIV strains. Therefore, close surveillance of resistance-associated mutations is essential. Drug resistance testing should be conducted prior to ART initiation, and early warning systems for drug resistance should be established to curb the spread of resistant variants.

Based on multivariate logistic regression analysis, we found that HIV-1 individuals aged 70 years or older had a higher clustering rate in the molecular transmission network. In recent years, the number of older people living with HIV in China has steadily increased, particularly among older men, who have emerged as a key population requiring urgent public health attention (42, 43). As an emerging high-risk group, older men appear to play a significant role in local HIV transmission. Among older men infected with HIV-1, we found that 9 individuals had both commercial heterosexual and homosexual behaviors, potentially facilitating the transmission of the virus to other men. In addition, some patients may further transmit HIV to their spouses. Previous studies suggested that older people often experience heightened psychological and emotional needs due to a lack of companionship and caregiving. As a result, some may seek extramarital sexual relationships for emotional or physical fulfillment (43). In rural areas, older men were among the main clientele of low-cost commercial sex venues. Those without stable sexual partners were particularly vulnerable to HIV infection (44). Moreover, a substantial proportion of older male clients had multiple sexual partners and report low rates of condom use, placing them in a bridging position for HIV transmission between female sex workers and lower-risk populations (45, 46). Furthermore, older people tended to have limited awareness of HIV and were less likely to access HIV-related counseling and testing services, contributing to the concealed spread of the virus (47).

These findings showed that older people and sex workers were at a higher risk of HIV transmission. Therefore, we suggested strengthening health education through online platforms and community-based programs to improve awareness of HIV prevention and ART among these vulnerable populations. Such efforts could promote early screening, diagnosis, and treatment adherence.

A total of eight large HIV-1 molecular clusters were identified, with all showing evidence of inter-city transmission. Notably, Cluster 1 was composed of individuals infected with subtype C, a finding rarely reported in previous studies. Most individuals in this cluster were older people who acquired HIV through heterosexual commercial sex. The two high-degree individuals were female sex workers, suggesting that a small number of sex workers may serve as transmission hubs by linking to multiple male clients. A similar transmission pattern was observed in a study conducted in Guangxi, where the largest cluster included two female sex workers and 18 older male clients (43). Four MSM were also identified in Cluster 1, suggesting the existence of hidden transmission chains involving bisexual behavior. This may account for the coexistence of heterosexual and homosexual transmission routes within the same cluster. Similar hybrid transmission patterns were also identified in Clusters 3, 4, and 6. Additionally, one older woman was identified in each of Clusters 3 and 5, both of whom were likely infected by spouses who had engaged in commercial sex. These observations highlight the risk of HIV transmission within older couples and reinforce the need to include them in targeted prevention strategies.

Clusters 2 and 7 were primarily composed of young MSM. Numerous studies had reported that young MSM play a central role in HIV-1 molecular transmission networks (18, 48). This population was often associated with multiple high-risk behaviors, including frequent partner change, participation in high-density sexual networks, engagement in receptive or versatile sexual roles, recreational drug use, low rates of HIV testing, unprotected anal intercourse, and lower levels of education (49). In our study, molecular transmission network analysis revealed that some MSM individuals concealed their same-sex sexual behaviors. This phenomenon may be attributed to the persistent stigma surrounding homosexuality in traditional Chinese culture. Due to fear of discrimination, MSM may be reluctant to disclose their sexual orientation, which may limit their access to HIV prevention and intervention services. Consequently, high-risk behaviors may be underreported or misclassified in epidemiological investigations (50, 51).

By constructing inter-regional intensity matrices of HIV-1 transmission links, the results showed that while intra-regional transmission remains the dominant mode of HIV-1 spread, inter-regional transmission should not be overlooked. Transmission intensity varied significantly across regions. Tongzhou exhibited the highest proportion of inter-regional transmission, possibly due to its large geographic coverage, adjacency to multiple districts, high population mobility, and active economic exchanges. In contrast, HIV-1 transmission in Rudong was largely localized,. Rudong, which had the highest level of population aging in Nantong, reported that people aged 60 years or older accounted for 42.93% of the population (52). Between 2021 and 2024, newly reported elderly HIV-1 subjects (≧50 years) account for 61.2% in Rudong. The aging of the population and the concentration of older HIV-1 infections may be important factors contributing to the higher intensity of local HIV-1 transmission compared with other regions.

Regarding subtype-specific spatial transmission, high levels of inter-regional spread of subtype C were observed among Chongchuan, Tongzhou, and Rugao, likely driven by two large clusters involving this subtype. Intra-city transmission rates for CRF01_AE and CRF55_01B were also relatively high (64.1% and 79.3%, respectively), suggesting these subtypes were more likely to spread locally. These patterns indicate that the geographic distribution of HIV-1 subtypes may be closely associated with regional demographic structure, mobility, and behavioral characteristics.

The findings of this study provided valuable evidence for tailoring region-specific and population-specific HIV prevention strategies. In areas such as Rudong, where transmission is primarily localized, priority should be given to community-based interventions targeting local high-risk groups. In contrast, in regions like Chongchuan, Tongzhou, and Rugao, where cross-district transmission is more active, it is crucial to strengthen inter-regional cooperation, enhance population mobility monitoring, and implement targeted interventions for transient and high-mobility populations to contain the spread of HIV-1 effectively.

This study has limitations. First, the molecular transmission network constructed was based solely on genetic similarity and does not reflect direct transmission relationships. While HIV molecular network analysis can reveal potential links between individuals living with HIV, it does not capture the full transmission pathway nor identify high-risk HIV-negative individuals within the network (53, 54). Second, the sequenced region was limited to the *pol* gene and did not include the integrase region; therefore, mutations conferring resistance to integrase inhibitors could not be analyzed. Third, molecular transmission network analysis alone cannot fully represent actual transmission dynamics. Its accuracy may be affected by sampling coverage and the timeliness of data collection. Thus, interpretations of molecular transmission networks should be combined with comprehensive epidemiological investigations for effective real-world application.

## Data Availability

The raw data supporting the conclusions of this article will be made available by the authors, without undue reservation.
