# Supplementary material for: Molecular transmission network characteristics and high-risk transmission analysis of newly reported HIV-1 subjects in Nantong, China
Source: Front Immunol. 2025 Dec 8;16:1716273. doi: 10.3389/fimmu.2025.1716273 (PMC12719490; doi:10.3389/fimmu.2025.1716273)
Supplement: Supplementary file 1 [file Table1.docx]

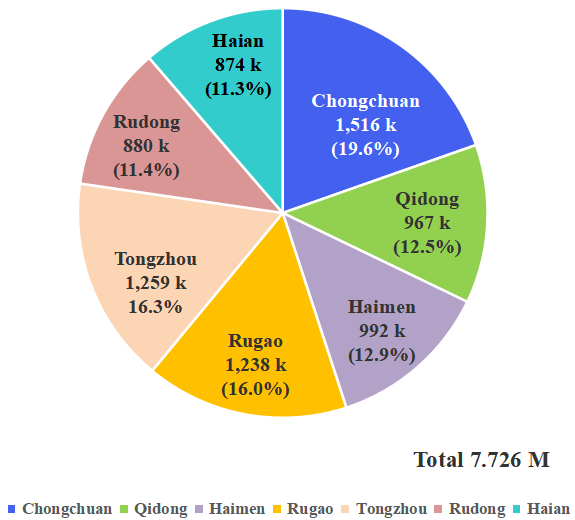
**Supplementary Figure 1.** Regional distribution of the resident population in Nantong, November 2020. The figure presents the total population and proportion of each district. Data were obtained from the Seventh National Population Census Bulletin of Nantong.
